# Polyphenols from Pulses: Recent Advances in Gut Health Benefits and Strategies to Elevate Their Concentrations

**DOI:** 10.3390/nu18121895

**Published:** 2026-06-11

**Authors:** Jiakai Wang, Rong Wei, Jiahong Wang, Ting Bai, Xinjie Jiang, Sumei Zhou, Dianzhi Hou

**Affiliations:** 1 Key Laboratory of Geriatric Nutrition and Health, Beijing Technology and Business University, Ministry of Education, Beijing 100048, China; 15003368165@163.com (J.W.); w17651727603@163.com (R.W.); 19801852052@163.com (J.W.); 18708015033@163.com (T.B.); zhousumei1001@163.com (S.Z.); 2 College of Food Science and Technology, Haidu College, Qingdao Agricultural University, Qingdao 266000, China

**Keywords:** pulse polyphenols, intestinal health, gut microbiota, enrichment techniques

## Abstract

Pulses are rich in diverse phenolic compounds, which play a vital role in human health. The biological activity of pulses’ polyphenols is highly dependent on their interaction with the gastrointestinal tract and gut microbiota. Polyphenols from pulses have been proven to be effective in the regulation of gut homeostasis, exerting antioxidant and anti-inflammatory effects, and alleviating various intestinal health problems caused by chronic diseases, thus receiving extensive attention. Furthermore, in view of the prebiotic potential of pulse polyphenols in gut health, they can be enriched by fermentation, germination, and physical-assisted technologies to realize their effective applications. In this review, we systematically summarize and analyze the phenolic compound profiles, which confer extensive gut health benefits, with emphasis on their potential enrichment strategies. A better understanding of polyphenols in pulses may open up new avenues for their application in the development of functional foods.

## 1. Introduction

As the edible seeds of *leguminous* plants, pulses are considered the second most important source of human food after cereals and occupy a significant position within the modern food nutrition system. Currently, the emerging scientific data support that the increased intake of pulses can reduce the risk of some chronic and metabolic diseases, which are closely associated with gut health [1,2]. Noticeably, the abundant phenolic compounds in pulses play an important role in the realization of their health benefits [3]. Pulse polyphenols can be used as prebiotics to regulate the gut microbiota and the intestinal barrier, thus maintaining the health of the host [4].

The bioaccessibility of pulse polyphenols, a critical determinant of their gut health benefits, begins in the stomach, where acidic conditions enhanced release. For example, the lentil hull polyphenols exhibited a 2.88-fold increase in total phenolic content during gastric digestion compared to the oral phase [5]. Further release occurred in the small intestine via pancreatic enzymes, while colonic fermentation converted indigestible polyphenols into absorbable small-molecule phenolic acids [6]. Concurrently, pulse polyphenols exerted prebiotic effects on gut microbiota by reshaping its composition. In vitro studies demonstrated that mung bean seed coat polyphenols increased the relative abundances of *Lactobacillus* and *Bacteroides* [7]. In vivo studies further confirmed that azuki bean seed-coat polyphenols significantly enriched the beneficial bacteria (*Akkermansia*) and suppressed the harmful *Proteobacteria* in colitis mice [8]. Additionally, they fortified intestinal barrier integrity, as evidenced by pea hull polyphenols, via upregulating tight junction proteins (claudin-1 and occludin, zonula occludens-1(ZO-1)) in Caco-2 cells [9]. In vivo, lentil and azuki bean polyphenols restored crypt architecture, increased goblet cells, and reduced permeability in colitis models [10]. Notably, pulse polyphenols exhibited potent antioxidant and anti-inflammatory activities. For instance, *C*-glycosylated flavonoids (vitexin and isovitexin) from mung beans effectively scavenged free radicals, contributing to antioxidant effects [11]; chickpea polyphenol extracts reduced pro-inflammatory cytokine expression by inhibiting the nuclear factor kappa-B (NF-κB) signaling pathway in cellular models [12]. Collectively, these pathways of action position pulse polyphenols as effective dietary strategies for preserving gut homeostasis.

Given the positive impact of phenolic compounds in pulses on gut health, in-depth exploration of their enrichment technologies is critical. Currently, the enrichment technologies primarily encompass fermentation, germination, and other physically assisted enrichment techniques. Fermentation primarily relies on hydrolytic enzymes secreted by microorganisms, such as β-glucosidase and xylanase, to break down bound phenolic compounds within the pulse matrix into free ones [13]. For instance, a recent study utilized Aspergillus awamori for the solid-state fermentation (SSF) of lentil, resulting in a maximum increase in total polyphenol content (TPC) of 122.8% for one cultivar [14]. Germination activated key enzymes in the phenylpropanoid pathway (phenylalanine ammonia-lyase, PAL; cinnamate 4-hydroxylase, C4H; and 4-coumarate-CoA ligase, 4CL) during seed sprouting, promoting the biosynthesis and release of polyphenols [15]. Van Hung et al. [16] demonstrated that mung beans germinated for 96 h under dark conditions exhibited a significantly higher TPC (7.19 mg FAE/g) than that of non-germinated seeds (3.82 mg FAE/g). Notably, under specific conditions, employing certain physical assistance techniques can further enhance polyphenol content. It was found that mung beans subjected to ultrasonic pretreatment followed by 48 h of germination exhibited a significantly higher TPC (7.63 mg/g) when compared with the conventional germination group (6.12 mg/g) [17].

Based on the aforementioned research background, this review was conducted using a systematic literature search in scientific databases such as Web of Science, Scopus, PubMed, and Google Scholar (2015–2025), with keywords including “pulse polyphenols,” “legume polyphenols,” “gut health,” “intestinal barrier,” “gut microbiota,” “fermentation,” “germination,” and “enrichment techniques.” The scope of the search was limited to eight major edible pulses (mung beans, azuki beans, common beans, broad beans, peas, chickpeas, lupins, and lentils), excluding oilseeds (such as soybeans and peanuts) and forage legumes. Studies addressing gut health-related indicators (such as microbial composition, intestinal barrier integrity, oxidative stress, and inflammation) or polyphenol enrichment strategies were included. Although previous studies have examined the effects of processing on pulse phytochemicals [18], a comprehensive synthesis specifically addressing the gut health benefits of pulse polyphenols, as well as a systematic summary of novel enrichment techniques, has yet to be performed. Therefore, this review aims to outline the effects of pulse polyphenols on gut health from the perspectives of regulating gut microbiota composition, improving intestinal barrier function, and exerting antioxidant and anti-inflammatory effects associated with gut homeostasis. Moreover, the technical advantages and influencing factors of novel enrichment techniques are compared. This review might provide researchers with a clear understanding of pulse polyphenols and facilitate further exploration of their potential applications.

## 2. Pulses and Their Polyphenols

Distinct from the broader concept of leguminous plants, pulses encompass dry common beans, dry peas, dry broad beans, chickpeas, lentils, lupins, cowpeas, and pigeon peas. Mung beans, azuki beans, and black-eyed peas were categorized under other unlisted dry beans [18]. This excludes immature fresh pods, such as green peas and broad beans, and does not include oilseeds like soybeans and groundnuts, or forage seeds like clover and alfalfa. Table 1 summarizes the major polyphenolic compounds and the TPC of the eight pulses.

**Figure 1:**
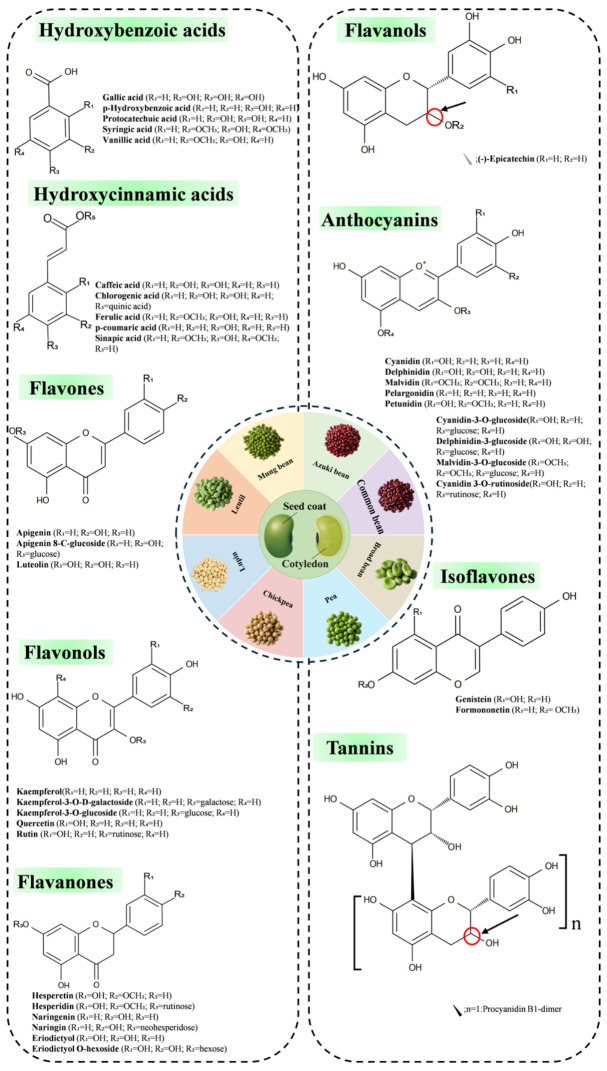
Common phenolic compounds in pulses.

Based on the chemical structures, polyphenolic compounds in pulses are primarily classified into phenolic acids (hydroxybenzoic acid and hydroxycinnamic acid), flavonoids (flavones, flavonols, flavanones, flavanols, anthocyanins, and isoflavones), and tannins (proanthocyanidins) (Figure 1). As shown in Table 1, the TPC in seed coats is significantly higher than that in cotyledons. In a study by Chaieb et al. [19], it was found that among 13 broad bean varieties, seed coats contributed up to 85.3% of the TPC. Similarly, 94.8% of the TPC in azuki beans was provided by their seed coat [20]. For different pulse varieties, significant variations in TPC are observed. Furthermore, within the same pulse variety, its seed coat color also exerts a substantial influence on TPC. Analysis of six different chickpea varieties cultivated under identical environmental conditions revealed that black-colored varieties exhibited a higher TPC (1.67 ± 0.01 mg GAE/g) than beige-colored varieties (1.23 ± 0.01 mg GAE/g) [21].

### 2.1. Phenolic Acids

Phenolic acids constitute critical secondary metabolites in pulses, categorized by chemical structure into hydroxycinnamic acids and hydroxybenzoic acids, which are widely distributed throughout pulses. Five hydroxybenzoic acids and nine hydroxycinnamic acids have been identified across eight pulses (Table 1). *p*-Coumaric acid, ferulic acid, sinapic acid, and caffeic acid are the most common hydroxycinnamic acids among the eight pulses, while gallic acid, *p*-hydroxybenzoic acid, protocatechuic acid, and eugenolic acid constitute the most common hydroxybenzoic acids. Hydroxycinnamic acids possess unsaturated side chains and typically occur in pulses as quinic acid esters or glycosides, whereas hydroxybenzoic acids feature simple benzene rings and are primarily present as glycosides. Significant variations are observed in predominant phenolic acids across different pulses. Mung beans predominantly contained high concentrations of bound caffeic acid, accounting for nearly 90% of total phenolic acids [11,22,23]. Azuki beans, conversely, exhibited ferulic acid and *p*-coumaric acid as predominant phenolic acids [20,24,25,26,27]. The phenolic acid profile of common beans was more complex, with dark-colored varieties rich in ferulic acid, sinapic acid, and their derivatives [28,29,30]. The content of eugenolic acid in broad beans was particularly notable, often being dozens of times higher than that of other phenolic acids [19,31,32]. The distribution of phenolic acids in peas exhibited tissue specificity, with the seed coat being rich in gallic acid and protocatechuic acid, often forming special conjugates with malic acid (*p*-coumaroyl malic acid) [33,34,35]. For lupin, the yellow ones were rich in protocatechuic acid, whereas the narrow-leaved ones primarily accumulated *p*-hydroxybenzoic acid [36,37,38]. The total phenolic acid content in lentils, along with specific components, such as ferulic acid, exhibited significant dependence on cultivar and geographical origin [39,40,41].

### 2.2. Flavonoids

Pulses contain six major flavonoid subclasses, namely flavones, flavonols, flavanones, flavanols, anthocyanins, and isoflavones (Table 1). The most abundant flavonoid compounds detected in eight pulses were flavones (vitexin, isovitexin, apigenin, and luteolin) and flavonols (quercetin, rutin, and kaempferol) (Table 1). Further analysis reveals that the composition and content of flavonoid compounds exhibit distinct variety-specific and tissue-distribution differences across different pulses. For example, among flavanols, mung beans were characterized by quercetin, kaempferol, and their glycosides (kaempferol-O-hexoside), which contributed to antioxidant activity [23]. Common beans were characterized by the presence of quercetin-O-glucosides and kaempferol-O-glucosides, with content also varying between varieties. Dark common bean-seeded varieties typically exhibited higher flavanol concentrations than light common bean-seeded ones [29]. Among flavones, vitexin and isovitexin constitute the core characteristic components in mung beans. As apigenin-type *C*-glycosides, the above compounds exhibited the highest concentrations in seed coats, reaching 37.43 mg/g and 47.18 mg/g, respectively [20]. Flavanone content was generally low, with only minor flavanone analogues detected in certain pea varieties and a few broad bean genotypes [32,33]. Among flavanols, catechin and epicatechin in azuki beans were concentrated in the seed coat, accounting for over 90% of the total flavanol content in the whole bean. Furthermore, geographical origin influenced flavanol content, with Canadian lentil varieties exhibiting higher concentrations than European cultivars [40]. Anthocyanins constituted a significant component of polyphenols, occurring exclusively in dark-colored pulse varieties. Common beans harbored 16 identified anthocyanins, predominantly in glycoside forms, including common delphinidin-3-glucoside and cyanidin-3-glucoside, which underpinned seed coat coloration and antioxidant activity [29]. Isoflavones, such as genistein and genistein aglycone derivatives, had only been found in lupins and chickpeas.

### 2.3. Tannins

Tannins in pulses primarily existed as condensed tannins, also known as proanthocyanidins or polymeric flavonoids, chiefly classified into types A and B. They commonly occur as dimers, trimers, and other polymeric forms. Among eight pulses, azuki beans exhibited the highest proanthocyanidin content at 97.42 mg CE/g [20]. These proanthocyanidins were predominantly distributed in the seed coat, with dark-colored varieties (black azuki beans) showing significantly higher concentrations than light-colored ones. Proanthocyanidins detected in common beans, broad beans, peas, and lentils were predominantly B-type dimers. Common beans were rich in proanthocyanidins (up to 57.13 mg/g in seed coats) [29]. For peas, proanthocyanidins were only found in the seed coats of dark-colored varieties [40,41]. Those in lentils also contained trimers besides B-type dimers. Notably, although proanthocyanidins were detected in mung beans and chickpeas, their specific types and degree of polymerization remained unclear [11,22,23,42]. For lupins, no tannins were detected.

## 3. Bioaccessibility of Pulse Polyphenols

The bioaccessibility of pulse polyphenols is a crucial prerequisite for their subsequent gut health benefits. Pulse polyphenols predominantly exist in the form of glycosides or ester-bound conjugates tightly associated with plant cell walls. These forms cannot be directly absorbed by the human intestine and must be converted into free forms through enzymatic hydrolysis in the gastrointestinal tract and microbial metabolism in the colon before they can be absorbed and exert their biological activities [43].

**Figure 2:**
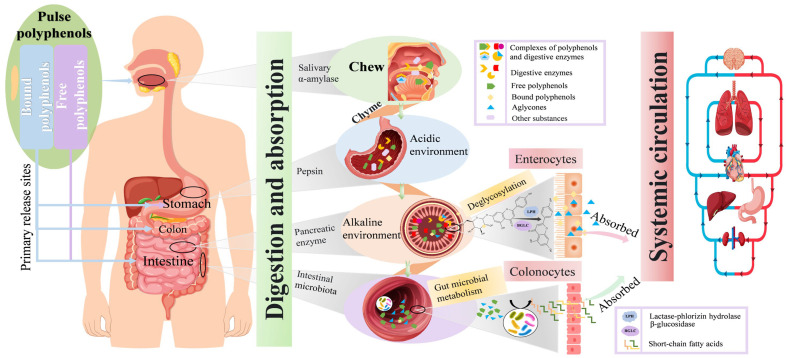
The digestion and absorption of pulse polyphenols in the human body.

Figure 2 illustrates the process of polyphenol digestion and absorption in the human body. The release of polyphenols from pulses in the oral cavity was negligible. For example, the TPC released from broad beans and common beans following simulated oral digestion accounts for only 3.65–9.22% of that released via acid or alkaline hydrolysis, indicating limited bioaccessibility [6]. Upon entering the stomach, the acidic environment created by gastric acid (pH 1.5–3.5) promotes the protonation of polyphenols. During gastric digestion, the bioaccessibility of TPC and total flavonoid content (TFC) in lentil hulls increased by 26.8% and 24.5%, compared to the oral phase [5]. Similarly, pea polyphenols also exhibited enhanced release during the gastric phase [44]. However, the profiles of polyphenols released by pulses at this stage were variety-dependent. Lupins primarily released apigenin derivatives in the gastric stage, with a release rate of up to 80%, whereas common beans mainly released phenolic acids (tannic acid and caffeic acid) [45,46]. Furthermore, most polyphenols were poorly absorbed in the stomach, with distinct behaviors among different classes exhibited. Phenolic acids, although partially released in the stomach, were structurally unstable [47]. In contrast, most flavonoids, such as quercetin, could resist gastric acid hydrolysis and remained poorly absorbed, with only limited absorption observed for certain flavonoid aglycones [48].

Currently, models for assessing the bioaccessibility of polyphenols in pulses are primarily categorized into small intestine models and colon models. Specifically, in the small intestine, the polyphenols that could be absorbed primarily included aglycones (via passive diffusion) and specific glycosides (via active transport). Aglycones typically had a smaller molecular weight, while glycosides (kaempferol tri-hexoside), despite having a larger molecular weight, could still be absorbed into the bloodstream via glucose transporters. For example, pea hull polyphenols reached their maximum concentrations during small intestinal digestion, with the main phenolic compounds released including quercetin glycosides and naringenin aglycone; simultaneously, TPC and TFC were 2.34-fold and 2.11-fold higher than at the gastric stage [44]. Similarly, following digestion in the small intestine, the TPC release rate of lentil polyphenols increased by 137% compared to the gastric phase, with the most significant increase observed in flavonol glycosides (up to 210%) [47]. Although pulse polyphenols were released in significant amounts in the stomach and small intestine, their overall bioaccessibility remained extremely low [49]. A significant portion of these polyphenols (flavonols, catechins, and proanthocyanidins) reached the colon, where they were metabolized by the gut microbiota and converted into more easily absorbed small-molecule phenolic acids (*p*-hydroxybenzoic acid, 3-hydroxybenzoic acid, and 4-hydroxybenzoic acid) [50]. The more significant role lay in the synergy between “microbial metabolism-metabolite absorption” and “microbiota structure regulation.” Research by Zhu et al. [6] indicated that colonic fermentation resulted in the release of polyphenols from broad beans equivalent to 80.0% of their alkali-hydrolyzed bound phenolic content. Similarly, the bioaccessibility of chickpea polyphenols peaked during the colonic phase [51]. Furthermore, the release of polyphenols from mung bean seed coats via colonic fermentation was significantly higher than that from conventional digestion and peaked at 12 h [52].

In summary, colonic fermentation plays a critical role in the bioaccessibility of pulse polyphenols. A large proportion of indigestible polyphenols (flavonols, catechins, and proanthocyanidins) reach the colon, where they are metabolized by the gut microbiota into more readily absorbable small-molecule phenolic acids, such as *p*-hydroxybenzoic acid and 3-hydroxybenzoic acid. This process not only enhances the release of bound polyphenols but also generates bioactive metabolites that could further interact with the gut environment.

Thermal processing can promote the release of polyphenols from the pulse matrix by breaking down cell walls, yet its impact on bioaccessibility during digestion is complex and method-dependent. For example, high-pressure cooking increased the TPC yield in broad beans by nearly fourfold, but after gastric digestion, their bioaccessibility was only 18–38%, and some flavonoids were undetectable [50]. Furthermore, after high-pressure cooking, the bioaccessibility of TPC in common beans in the small intestine increased to approximately 50% compared to raw beans [53]. However, further frying reduced bioaccessibility to about 30%, which was lower than that of cooked pulses that had not been fried. In another study, after steaming, black chickpeas showed the 35.1% increase and 72.9% decrease in TPC and TFC compared to raw beans; in brown lentils, the TPC and TFC decreased by 7.4% and 57%, respectively [51]. This suggested that heat treatment may reduce the dose of flavonoids reaching the colon, thereby affecting their potential for metabolism by the gut microbiota.

Accordingly, relying solely on in vitro digestion results from raw pulses may overestimate or underestimate the bioaccessibility of polyphenols following actual consumption of cooked pulses. When studying the health benefits of pulse polyphenols, it is crucial to use cooked pulse samples that more closely reflect actual consumption patterns and to systematically evaluate the effects of different processing methods to accurately assess the bioaccessibility of pulse polyphenols.

**Table 1:** The main phenolic compounds in pulses.

Sample	Part	TPC	Major Phenolic Compounds	References
Mung bean (*Vigna radiata* L.)	Cotyledon	0.59 mg GAE/g	N.M.	[11,22,23,54,55]
	Seed coat	29.58 mg GAE/g	Hydroxybenzoic acids: syringic acid; Hydroxycinnamic acids: caffeic acid, *p*-coumaric acid, ferulic acid; Flavones: vitexin, isovitexin, apigenin derivatives; flavonols: kaempferol-O-hexoside, kaempferol-O-rutinoside; flavanols: catechin-O-hexoside	
Azuki bean (*Vigna angularis* Wild.)	Cotyledon	0.40 mg GAE/g DW	N.M.	[20,24,25,26,27]
	Seed coat	54.45 mg GAE/g DW	Hydroxybenzoic acids: protocatechuic acid, gallic acid; Hydroxycinnamic acids: *p*-coumaric acid, ferulic acid, sinapic acid; Flavones: *vitexin*, *isovitexin*; flavonols: *quercetin*, *rutin*; flavanols: (+)-*catechin*, (+)-*epicatechin*; anthocyanins: *cyanidin*; tannin: procyanidin B1, procyanidin B3, catechiopyranocyanidin A, catechiopyranocyanidin B, vignacyanidin	
Common bean (*Phaseolus vulgaris* L.)	Cotyledon	N.M.	Hydroxycinnamic acids: *p*-coumaric acid, ferulic acid, cinnamic acid	[28,29,30,56]
	Seed coat	0.25–35.11 mg GAE/g	Hydroxybenzoic acids: protocatechuic acid; Hydroxycinnamic acids: *p*-coumaric acid, ferulic acid, sinapic acid; Flavonols: quercetin-3-O-rutinoside, kaempferol-3-O-glucoside; isoflavones: genistein; flavanones: eriodictyol-O-hexoside 1, eriodictyol-7-O-glucoside, hesperetin-O-glucuronyl-hexoside, naringenin derivative, eriodictyol-O-acyl hexoside; flavanols: epicatechin, catechin; anthocyanins: delphinidin-3-glucoside, cyanidin-3-glucoside, petunidin-3-glucoside, pelargonidin-3-glucoside, malvidin-3-glucoside; tannin: procyanidin B2	
Broad bean (*Vicia faba* L.)	Cotyledon	15.89–27.21 mg GAE/g DW	Hydroxybenzoic acids: syringic acid, ferulic acid (trans); Hydroxycinnamic acids: ferulic acid (trans), chlorogenic acid; Flavanols: catechin	[19,31,32]
	Seed coat	45.60–107.65 mg GAE/g DW	Hydroxybenzoic acids: protocatechuic acid, *p*-hydroxybenzoic acid, syringic acid, ferulic acid (trans), vanillic acid; Hydroxycinnamic acids: *p*-coumaric acid; Flavones: vitexin; flavonols: rutin; flavanols: epicatechin, catechin gallate; tannin: prodelphinidin dimer, gallate prodelphinidin dimer, digallate procyanidin dimer, procyanidin gallate	
Pea (*Pisum sativum* L.)	Cotyledon	N.M.	Hydroxybenzoic acids: *p*-hydroxybenzoic acid; Hydroxycinnamic acids: ferulic acid, *p*-coumaric acid (trans); Flavones: apigenin-8-C-glucoside; flavonols: quercetin-3-O-galactoside; flavanols: epigallocatechin	[33,34,35,57]
	Seed coat	2.78–46.56 mg GAE/g	Hydroxybenzoic acids: gallic acid, protocatechuic acid, *p*-hydroxybenzoic acid; Hydroxycinnamic acids: ferulic acid, *p*-coumaric acid (trans); Flavones: luteolin glycoside, apigenin-8-C-glucoside, apigenin, luteolin; flavonols: quercetin-3-O-galactoside, kaempferol-3-robinoside-7-rhamnoside; flavanones: naringin, naringenin; flavanols: epigallocatechin, catechin, epicatechin; anthocyanins: pelargonidin-3-(6″-p-coumarylglucoside)-5-(6‴-acetylglucoside); tannin: dimer of prodelphinidin, dimer of prodelphinidin	
Chickpea (*Cicer arietinum* L.)	Cotyledon	15.24 mg GAE/g DW	N.M.	[12,58]
	Seed coat	16.52–75.94 mg GAE/g DW	Hydroxybenzoic acids: *p*-hydroxybenzoic acid, vanillic acid, gallic acid, protocatechuic acid; Hydroxycinnamic acids: ferulic acid, *p*-coumaric acid; Flavonols: rutin; isoflavones: genistein; anthocyanins: cyanidin, petunidin, delphinidin	
Lupin (*Lupinus angustifolius* L.)	Cotyledon	N.M.	Hydroxybenzoic acids: protocatechuic acid, *p*-hydroxybenzoic acid; Hydroxycinnamic acids: caffeic acid, *p*-coumaric acid; Flavones: apigenin-6,8-di-C-β-glucopyranoside; isoflavones: genistein	[36,37,38]
	Seed coat	0.68–1.28 mg GAE/g DW	Hydroxybenzoic acids: protocatechuic acid; Hydroxycinnamic acids: cinnamic acid glucoside, dicaffeoylquinic acid; flavones: apigenin-7-O-β-glucopyranoside; isoflavones: genistein	
Lentil (*Lens culinaris* Medik.)	Cotyledon	4.56–8.34 mg GAE/g	Hydroxybenzoic acids: *p*-hydroxybenzoic acid; Hydroxycinnamic acids: *p*-coumaroyl malic acid (trans); Flavonols: kaempferol triglycoside, quercetin-3-glucoside; isoflavones: formononetin; flavanols: catechin gallate	[39,40,41,59,60]
	Seed coat	30.6–70.37 mg GAE/g	Hydroxybenzoic acids: *p*-hydroxybenzoic acid, syringic acid, gallic acid; Hydroxycinnamic acids: *p*-coumaric acid (trans), ferulic acid; Flavones: luteolin-7-O-(2-apiosyl-glucoside); flavonols: kaempferol-3-glucoside, quercetin-3-xyloside, kaempferol tetraglycoside; flavanones: hesperidin; flavanols: (+)-catechin-3-O-glucoside, procyanidin dimers, epicatechin gallate; anthocyanins: delphinidin-3-O-sambubioside, cyanidin-3-O-rutinoside; tannin: procyanidin dimers, digallate procyanidin dimer, prodelphinidin trimer, digallate procyanidin trimer, procyanidin tetramer, prodelphinidin tetramer	

N.M.: not mentioned.

## 4. The Benefits of Pulse Polyphenols for Gut Health

Numerous in vitro and in vivo studies have robustly demonstrated the positive effects of pulse polyphenols on gut health. Moreover, they can not only regulate gut microbiota disorders and enhance the functional barrier of the intestine, but also exert antioxidant and anti-inflammatory effects, maintain intestinal mucosa integrity, and bolster gut immune function.

### 4.1. Regulation of Gut Microbiota

Disruption of the gut microbiota is closely associated with the occurrence and development of chronic diseases. In vitro and in vivo studies demonstrated that pulse polyphenols significantly reshaped the gut microbiota composition and increased beneficial metabolites, which ultimately restored gut homeostasis [43,61].

Beyond these general effects, the modulation of gut microbiota by pulse polyphenols is selective and variety-dependent. For instance, an in vitro study demonstrated that polyphenols from different common bean varieties selectively modulated gut microbiota [62]. Black common bean polyphenols promoted the growth of *Lachnospira*, *Ruminococcaceae*, and *Terrisporobacter* species, whereas light gray common bean polyphenols enhanced short-chain fatty acid-producing *Roseburia* species. Table 2 summarizes the gut microbiota components modulated by pulse polyphenols in both in vitro and in vivo studies.

#### 4.1.1. In Vitro Studies

An in vitro digestion study of flavonoids (quercetin and catechin) and phenolic acids (vanillic acid and *p*-hydroxybenzoic acid) from mung bean seed coat demonstrated that after 12 h of simulated colonic fermentation, the relative abundances of *lactobacilli* and *bacteroides* increased to 2.61% and 8.83%, respectively [52]. Concurrently, the intestinal pH decreased from an initial 6.8 to 4.9, selectively inhibiting the growth of potential pathogens, such as *Shigella*. In another study, the effects of single phenolic acid (gallic acid) and a single flavone (vitexin) versus a polyphenolic complex (rich in flavonoids and phenolic acids) on gut microbiota were compared [1]. The polyphenolic complex group exhibited targeted suppression of *Escherichia-Shigella* abundance and *Klebsiella* proliferation, whereas the single-polyphenol groups showed no significant inhibitory effect on them. However, the regulation of gut microbiota by pulse polyphenols extends beyond structural alterations to directly modulate microbial metabolic functions. Azuki bean polyphenol extract (AE), which was rich in anthocyanins and proanthocyanidins, significantly reduced the ammonia nitrogen production and concentration in the fermentation system (*p* < 0.01), while optimizing fermentation pH [63]. Notably, the relationship between pulse polyphenols and gut microbiota is not unidirectional regulation but rather bidirectionally interactive. Hydrolytic enzymes released by gut microbiota are pivotal for the release of bound polyphenols. Chickpea flavanone glycosides (hesperetin 5,7-O-diglucuronide) and isoflavone derivatives (glycitein 7-O-glucuronide) reshaped microbial communities by selectively screening dominant metabolic strains of *Escherichia*, *Klebsiella*, *Bacteroides*, and *Veillonella*, which enhanced the release of bound polyphenols [64].

#### 4.1.2. In Vivo Studies

Mung bean flavones (vitexin and isovitexin) significantly altered gut microbiota composition in a type 2 diabetes mellitus (T2DM) mouse model, evidenced by the decreased abundance of the phyla *Firmicutes* and *Proteobacteria*, and increased abundance of *Bacteroidetes* [7]. Similarly, in another study, compared to the de-polyphenolized group, refilling the azuki bean seed coat with bound polyphenols (mainly proanthocyanidins and flavonols) for one week significantly restored the gut microbiota structure, enriched the beneficial bacteria (*Verrucomicrobia*), and suppressed harmful *Proteobacteria*, thereby alleviating colonic inflammation [8]. Sun et al. [65] employed α-diversity and β-diversity analyses to demonstrate that intervention with black common bean husk anthocyanins (predominantly cyanidin-3-O-glucoside) in a T2DM rat model significantly altered microbial abundance and compositional structure. Moreover, the intervention dosage of pulse polyphenols directly influenced the regulatory effects on gut microbiota. It was found that administering high and low doses of azuki bean polyphenol water extract (AWE) to high-fat-induced mice significantly suppressed the harmful bacteria (*Blautia*, *Ruminococcus*) in both groups, but only the high-dose group exhibited increased *Lactobacillus* abundance [66]. Similarly, after 14 d of intervention with pea flavonols (quercetin derivatives) and flavanols (catechin) in colitis-induced mice, only the high-dose group significantly reversed gut microbiota imbalance, promoting short-chain fatty acid (SCFA)-producing bacteria (*Lacobacillaceae* and *Lachnospiraceae*) while suppressing pro-inflammatory *Paraprevotellaceae* [9]. However, distinct pathogenic bacteria exhibited markedly divergent responses to polyphenol concentrations. Following administration of high-dose, medium-dose, and low-dose AWE to colitis-induced mice, *Escherichia* coli was suppressed only in the low-dose group, while the *Bacteroides* showed dose-dependent inhibition [67]. Furthermore, chickpea isoflavones (biochanin A, genistein, and formononetin) not only restored gut microbiota dysbiosis but also elevated SCFA levels in the caecum, with the most pronounced changes occurring in acetate, propionate, and butyrate [68]. Specifically, it enriched beneficial bacteria such as *Enterococcaceae* and *Bacteroidaceae*, while suppressing the pathogenic bacterium *Corynebacterium*. Notably, processing methods significantly influence the regulatory effects of pulse polyphenols on gut microbiota. Acid hydrolysis improved proanthocyanidin effects on gut microbiota. It restored diversity and homeostasis by degrading polymers into absorbable monomers, unlike the unhydrolyzed group [69]. Following intervention with fermented broad bean polyphenol extract (rich in phenolic acids, flavonols, and flavones) in mice, both the Shannon index and the relative abundance of specific beneficial bacteria were significantly higher than in the unfermented group [3].

**Table 2:** Effects of pulse polyphenol extracts on the gut microbiota in in vitro and in vivo studies.

In Vitro/In Vivo Studies	Model	Polyphenols Sources	Dosages	Key Findings	Reference
In vitro studies	In vitro simulated digestion and colonic fermentation	Mung bean seed coat polyphenols extract	N.M.	Phylum level: ↑ *Bacteroidetes*, ↓ *Firmicutes*, *Proteobacteria*, *Actinobacteria* Genus level: ↑ *Lactococcus*, *Bacteroides*, ↓ *Escherichia coli*, *Shigella*	[52]
	A mixed culture of pig fecal bacteria	Azuki bean polyphenols extract	1% (*w*/*v*)	Phylum level: ↑ *Verrucomicrobia*, *Actinobacteria*, *Bacteroidetes*, ↓ *Firmicutes*, *Proteobacteria* Genus level: ↑ *Akkermansia*, *Bifidobacterium*, *Bacteroides*, *Lactobacillus*, *Faecalibacterium*, *Roseburia*, ↓ *Clostridium*, *Escherichia*, *Shigella*	[63]
In vivo studies	T2DM mice	Germinated mung bean polyphenols	100–150 mg/kg	Phylum level: ↑ *Bacteroidetes*, ↓ *Firmicutes*, *Proteobacteria* Genus level: N.M.	[7]
	DSS-induced acute murine colitis	Azuki bean seed coat polyphenols	500 mg/kg	Phylum level: ↑ *Verrucomicrobiota*, ↓ *Proteobacteria*, *Bacteroidetes* Genus level: ↑ *Akkermansia*	[8]
	High-fat-diet-induced obese mice	Azuki bean hot water extract	5% (*w*/*w*)	Phylum level: ↑ *Bacteroidetes*, ↓ *Firmicutes* Genus level: ↑ *Lactobacillus*, *Akkermansia*, ↓ *Anaerotruncus colihominis*, *Butyrivibrio proteoclasticus*	[66]
	T2DM mice	Black common bean husk anthocyanin extract	N.M.	Phylum level: ↑ *Verrucomicrobia*, *Firmicute*, ↓ *Actinobacteria* Genus level: ↑ *Akkermansia*, *Coprococcus*, *Bacteroides*, *Lachnobacterium*, ↓ *Bifidobacterium*	
	Healthy mice	Unfermented faba bean polyphenol-enriched extract	250 mg/kg	Phylum level: ↑ *Bacteroidota*, *Proteobacteria*, *Deferribacteres*, ↓ *Firmicutes* Genus level: ↑ *Lactobacillus*, *Helicobacter*, ↓ *Butyrivibrio* sp.	[3]
	DSS-induced colitis mouse	Green pea hull polyphenol extracts	600 mg/kg	Phylum level: ↑ *Firmicutes*, *Proteobacteria*, ↓ *Bacteroidetes*, *Verrucomicrobia* Genus level: ↑ *Lactobacillu*, ↓ *Akkermansia*	[9]
	High-fat-diet-induced obese mice	Pea polyphenolics	N.M.	Phylum level: ↑ *Bacteroidetes*, ↓ *Firmicutes* Genus level: ↑ *Turicibacter*, *Oscillospira*, ↓ *Lactobacillus*, *Bifidobacterium*, *Dehalobacterium*, *Allobaculum*	[69]
	High-fat diet and streptozotocin-induced type 2 diabetic rats	Chickpea polyphenols extract	3 g/kg	Phylum level: ↑ *Bacteroidetes*, *Verrucomicrobia*, ↓ *Firmicutes*, *Actinobacteria* Genus level: ↑ *Bacteroides*, *Parabacteroides*, *Blautia*, *Enterococcus*, *Sutterella*, *Turicibacter*, *Dorea*, *Akkermansia*, ↓ *Corynebacterium*, *Ruminococcus*, *Allobaculum*, *Facklamia*, *Lachnobacterium*	[68]

N.M.: not mentioned; ↑: pulse polyphenols-increased bacterial strains; ↓: pulse polyphenols-decreased bacterial strains.

### 4.2. Improvement of Intestinal Barrier

Reduced expression of tight junction proteins and thinning of the mucus layer can permit endotoxins, pathogens, and their metabolites to enter the bloodstream, thereby inducing metabolic disorders, accompanied by excessive activation of the local intestinal immune system. However, extensive in vitro and in vivo studies have confirmed that pulse polyphenols can synergistically repair the intestinal barrier through multiple pathways [4,8]. These mechanisms include promoting mucin secretion by goblet cells to enhance mucus layer defense, regulating tight junction protein expression to reduce intestinal permeability, suppressing inflammatory cytokines to alleviate local inflammation, and further consolidating barrier function. Table 3 summarizes the effects of pulse polyphenols on the intestinal barrier mediated by the aforementioned mechanisms.

#### 4.2.1. In Vitro Studies

Currently, extensive in vitro studies using the Caco-2 cell model have demonstrated that pulse polyphenols can enhance intestinal barrier function. The in vitro digestive product of pea flavonols (quercetin derivatives) and flavanols (catechin conjugates) significantly inhibited the inflammatory response in lipopolysaccharide (LPS)-stimulated RAW264.7 cells while substantially enhancing the protein expression of claudin-1, occludin, and ZO-1 in Caco-2 cells, which effectively preserved intestinal barrier integrity by maintaining transepithelial electrical resistance (TEER) values [44]. In another study, when lentil flavonols (kaempferol derivatives) and proanthocyanidins (epicatechin) were applied to Caco-2 cells under IL-1β-induced inflammatory conditions, they significantly increased the gene expression of occludin and cadherin-1 (CDH1) and elevated the occludin and CDH1 protein levels [70]. Similarly, red lentil flavonols extract at 150 μg/mL concentration significantly reduced LPS-induced permeability in Caco-2 cell monolayers and promoted the tight junction protein expression, thereby improving the intestinal barrier integrity [4]. Notably, suppressing the production of inflammatory cytokines is crucial for improving the intestinal barrier. However, current in vitro studies regarding the effects of pulse polyphenols on the intestinal barrier remain limited, as it primarily stems from studies on peas, lentils, and common beans. Therefore, further research is required to explore a broader range of pulse varieties and validate the efficacy of their polyphenolic compounds in enhancing intestinal barrier function.

#### 4.2.2. In Vivo Studies

Consistent with in vitro research results, emerging findings of in vivo studies have also confirmed the effectiveness of pulse polyphenols in improving the intestinal barrier function. Researchers administered mung bean seed coat-derived polyphenols (phenolic acids, flavones, and flavanols) to a colitis mouse model, indicating that they restored crypt architecture, increased goblet cells to protect the mucus layer, suppressed pro-apoptotic proteins, and maintained epithelial cell homeostasis, while the polyphenol-removed group showed no such effects [10]. Specifically, these phenolic acids, flavones, and flavanols promoted goblet cell proliferation and mucin secretion, thereby reinforcing the mucus layer as the first line of defense for intestinal mucosal integrity. Mazewski et al. [71] found that treatment of mice with colon cancer with a water extract of black lentil polyphenols (rich in anthocyanins, flavonols, and proanthocyanidins) significantly reduced the colonic mucosal injury scores and inhibited the goblet cell loss. Similarly, bound polyphenols from azuki bean seed coats yielded identical effects in restoring barrier integrity [8]. Compared with the de-phenolized dietary fiber group, the backfilled polyphenol group significantly promoted the goblet cell proliferation and mucin secretion and reduced the serum levels of intestinal barrier damage markers and LPS. The regulation mechanism was related to the upregulated occludin, claudin1, and ZO-1 expression at both the gene and protein levels, which directly contributed to the maintenance of intestinal mucosal integrity by reducing epithelial permeability. In addition, Guo et al. [72] found that pea flavonols and flavanol extract significantly maintained barrier function in dextran sulfate sodium (DSS)-induced NAFLD mice by reducing the serum D-mannitol and LPS levels and upregulating the colonic claudin-1 expression, thereby reducing the entry of harmful substances into the bloodstream. Notably, the intervention dose of pulse polyphenols directly influenced the extent of restoration of intestinal barrier integrity. Following AWE intervention in colitis mice, only the high-dose and medium-dose groups exhibited significantly prolonged colon length and markedly reduced ileal permeability [67]. Similarly, green pea hull polyphenol extract (rich in flavonols, flavanols, and proanthocyanidins) improved the intestinal barrier integrity in colitis mice in a dose-dependent manner, with the high dose (600 mg/kg) exerting significant and comprehensive repair effects [9].

In addition to strengthening the physical barrier, certain pulse polyphenols have been shown to enhance the intestinal immune barrier. Studies indicated that azuki bean flavonoid and phenolic acid extract significantly increased mucin levels in the rat cecum, and the mucus layer formed by mucin served as the first line of defense for the intestinal immune barrier [63]. Furthermore, in an immunosuppressed mouse model, Liu et al. [73] demonstrated that mung bean polyphenol extract (rich in proanthocyanidin, flavone, flavanone, flavonol, and isoflavone) bolstered gut immune functions by activating the mitogen-activated protein kinase (MAPK) signaling pathway and enhancing phosphorylation of P38, ERK, and JNK, which in turn upregulated intestinal cytokine expression (IL-2 and IFN-γ) and improved immune-related gene transcription. Collectively, these findings suggested that pulse polyphenols could enhance the immune defense capacity of the intestinal mucosa at multiple levels by reinforcing the mucus layer and activating immune-related signaling pathways, thereby more comprehensively maintaining intestinal barrier integrity.

### 4.3. Antioxidant Activity Associated with Gut Health

Oxidative stress damages the intestinal mucosa and disrupted redox balance, both closely linked to chronic intestinal diseases. The in vitro and in vivo studies confirmed that pulse polyphenols alleviated intestinal oxidative stress by increasing antioxidant enzyme activities and lowering oxidative damage markers [74,75,76].

#### 4.3.1. In Vitro Studies

In a heat-stress-induced Caco-2 cell damage model, mung bean flavones and phenolic acids upregulated genes involved in oxidative phosphorylation and reduced ROS accumulation [77]. Similarly, after intervention in an AAPH-induced oxidative stress model in Caco-2 cells using black common bean anthocyanins (delphinidin-3-glucoside) obtained via supercritical CO_2_ extraction, a significant positive correlation was observed between total phenolic content and cellular antioxidant activity (CAA), with inhibition rates reaching up to 74.7% [78]. Common bean flavanols and flavonols were shown to possess significant antioxidant activity not only in Caco-2 cells but also in HT-29 cells [79]. It has been demonstrated that common bean polyphenols inhibited hydroxyl radical-induced DNA degradation and effectively blocked the oxidation of LDL. In addition, the antioxidant activity of pulse polyphenols in the intestine exhibited a dose-dependent relationship. Chen et al. [80] found in Caco-2 cells that common bean flavanols (catechin and epicatechin) and phenolic acids can enhance the activity of SOD, CAT, GPx, and GR, as well as levels of reduced glutathione (GSH), with the high-dose group (100 μg/mL) showing superior effects compared to the low-dose group (50 μg/mL), thereby exerting antioxidant effects by regulating antioxidant signaling pathways in intestinal epithelial cells.

**Table 3:** Effects of pulse polyphenols on gut barrier integrity in in vitro and in vivo studies.

In Vitro/In Vivo Studies	Model	Polyphenols Sources	Dosages	Key Findings	Reference
In vitro	Caco-2 cells	Phenolic-rich cranberry bean extract	500 μg/mL	↓ IL-8 production	[80]
	Caco-2 cells	Pea seed hull polyphenols	900 μg/mL	↑ Claudin-1, occludin, and ZO-1 expression ↑ TEER	[44]
	Caco-2 cells raw264.7	Pea seed hull polyphenols	400 μg/mL	↓ IL-6, TNF-α production ↓ iNOSmRNA expression, NO production	
	IL-1β stimulated Caco-2 cells	Lentil phenolic compounds	N.M.	↑ CDH-1, occludin expression ↓ IL-6, IL-8 production	[70]
	Caco-2 cells	Red lentil hull extract	150 μg/mL	↑ Claudin-1, occludin expression	[4]
In vivo	**CDH1** -induced male C57BL/6 mice	Mung bean seed coat polyphenols	N.M.	↑ Claudin-1, occludin, ZO-1 expression, and IL-10 production ↓ Bax, Caspase-3, Caspase-9 expression, IL-6, TNF-α production	[10]
	DSS-induced male C57BL/6 mice	Azuki bean seed coats polyphenols	500 mg/kg	↑ Claudin-1, occludin, ZO-1 expression, and goblet cells	[8]
	DSS-induced male C57BL/6 mice	Pea hull polyphenols	600 mg/kg BW/d	↑ Claudin-1, occludin, and ZO-1 expression ↓ IL-6, TNF-α, and IL-β production	[9]
	AOM/DSS-induced colitis-associated colon cancer mouse	Black lentil polyphenols water extract	600 mg/kg	↑ Goblet cells, colonic mucosal	[71]

N.M.: not mentioned; ↑: increase following pulse polyphenol intervention; ↓: decrease following pulse polyphenol intervention.

#### 4.3.2. In Vivo Studies

In vivo studies demonstrated that in a T2DM mouse model, mung bean flavones extract not only reshaped the gut microbiota but also significantly elevated serum SOD and GSH-Px activities and reduced malondialdehyde (MDA) levels [7]. Similarly, bound polyphenols (flavonols and flavanols) from azuki bean seed coat polyphenols lowered colonic oxidative stress markers in colitis-induced mice by modulating gut microbiota composition [8]. Furthermore, green pea flavonols and flavanols extract significantly improved SOD and GSH-Px activities and reduced MDA levels in the colon of colitis-induced mice by enhancing intestinal barrier function, thus reducing oxidative damage [9,74].

### 4.4. Anti-Inflammatory Activity Associated with Gut Health

Inflammation is a defensive response to harmful stimuli, but its sustained activation could lead to chronic diseases such as colitis and metabolic syndrome. Pulse polyphenols lowered pro-inflammatory cytokine levels, inhibited nitric oxide (NO) and inducible nitric oxide synthase (iNOS)/cyclooxygenase-2 (COX-2) production, and suppressed NF-κB and MAPK signaling pathways, thereby alleviating intestinal and systemic inflammation [4,8].

#### 4.4.1. In Vitro Studies

In the co-culture models that mimicked the intestinal microenvironment, it was found that pulse polyphenols significantly suppressed pro-inflammatory factor production while enhancing barrier integrity. For example, in a Caco-2/RAW264.7 co-culture system, green pea flavonols and flavanols significantly reduced LPS-induced TNF-α, NO, and IL-6 levels and enhanced tight junction protein expression and transepithelial electrical resistance [44]. Similarly, chickpea and lentil polyphenols achieved comparable effects in this model [12,47]. Additionally, Peng et al. [4] used lentil flavonols and phenolic acids in an HT-29 colon cell model to demonstrate, for the first time, that NF-κB and nuclear factor erythroid 2-related factor 2 (Nrf2) exhibited mutually inhibitory negative feedback crosstalk. Furthermore, the anti-inflammatory effects of pulse polyphenols in the gut were dose-dependent. Chen et al. [80] found in Caco-2 cells that the high-dose group (500 μg/mL) of common bean polyphenols (flavanols, proanthocyanidins, and phenolic acids) showed greater efficacy in inhibiting TNF-α-induced IL-8 secretion than the low-dose group (50 μg/mL), thereby exerting anti-inflammatory effects by modulating inflammatory signaling pathways in intestinal epithelial cells.

#### 4.4.2. In Vivo Studies

Animal studies further confirmed that in a DSS-induced colitis mouse model, mung bean seed coat polyphenols restored crypt architecture, increased goblet cell numbers, and concurrently reduced colonic IL-6 and TNF-α production [10]. Similarly, bound polyphenols (flavonols and flavanols) from azuki bean seed coat significantly lowered serum LPS and pro-inflammatory cytokine (IL-1β, IL-6, and TNF-α) levels [8]. In a diabetic rat model, chickpea isoflavones and flavonols extract reduced inflammation-related markers by restoring gut microbiota dysbiosis and elevating SCFA levels [68]. However, current in vivo studies indicated that the anti-inflammatory activity of pulse polyphenols was not limited to reducing inflammatory factor levels. Most studies confirmed that they could ameliorate metabolic diseases via the “gut–liver axis” (or gut microbiota–systemic axis) pathway. In an NAFLD mouse model, pea flavonols and flavanols extract inhibited hepatic Toll-like receptor 4 (TLR4)/NF-κB signaling while upregulating colonic tight junction proteins and decreasing serum LPS levels [72]. Furthermore, mung bean polyphenols strengthened the intestinal immune barrier in immunocompromised mice by activating the MAPK signaling pathway and upregulating tight junction proteins, thereby alleviating systemic inflammation [73]. Notably, the anti-inflammatory effects of pulse polyphenols were typically dose-dependent [67].

It remained unclear whether the doses of pulse polyphenols used in animal models in this review (100–3000 mg/kg/day) were feasible for humans. When adjusted for body surface area, these doses corresponded to human equivalent doses [81] of 8–243 mg/kg/day, equivalent to 0.5–14.6 g/day for a 60 kg adult [82]. Such amounts far exceeded the concentrations provided by typical dietary consumption of whole pulses. A standard serving (200 g cooked pulses) delivered only 0.2–0.5 g of total polyphenols [83]. Therefore, it was unrealistic to directly translate these supradietary doses into recommended human intake concentrations. Further clinical studies were needed to bridge this gap [84].

In vitro and in vivo studies have demonstrated that pulse polyphenols regulate gut microbiota, improve intestinal barrier, and exert antioxidant and anti-inflammatory effects. Flavonoids (flavonols, catechins, and procyanidins) and phenolic acids were the major bioactive constituents [85]. However, most studies treated these mechanisms, microbiota modulation, barrier protection, oxidative stress alleviation, and inflammation suppression, as separate phenomena. Few have systematically investigated whether polyphenols mediate barrier repair or anti-inflammatory responses through specific microbial regulation or crosstalk between redox and immune pathways [69,86]. Moreover, the specific compounds responsible for each effect remain unclear, and structure–activity relationships are poorly defined. Future research should expand to more pulse varieties and investigate individual phenolic compounds, particularly their synergistic actions in the gut.

## 5. Enrichment Technologies

**Figure 3:**
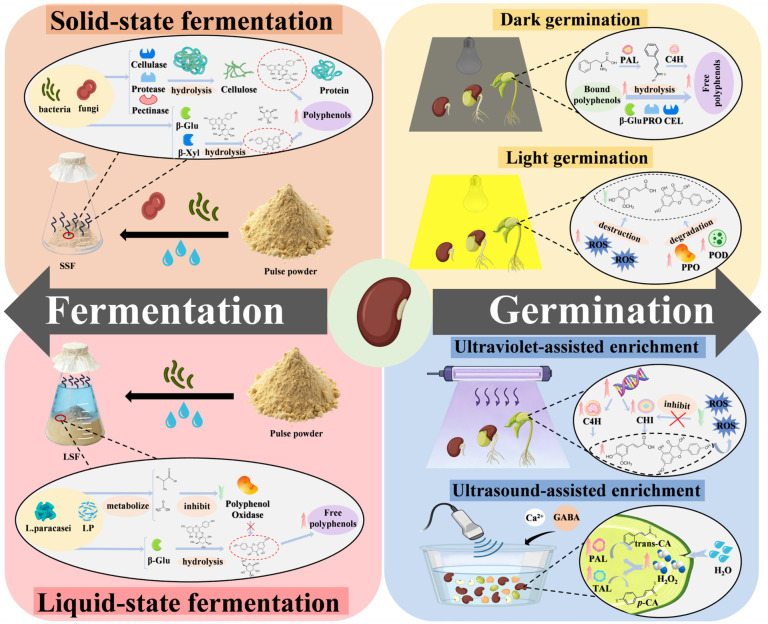
The intrinsic mechanisms of polyphenol fermentation and germination in pulses.

The efficient enrichment of pulse polyphenols is crucial for enhancing their content and biological activity. Currently, enrichment technologies are primarily categorized into fermentation, germination, and other physically assisted enrichment techniques (such as ultrasonication and ultraviolet stress). Table 4 summarizes the effects of different enrichment technologies on pulse polyphenol content. Figure 3 reveals the underlying mechanisms of fermentation and germination enrichment.

### 5.1. Fermentation Enrichment

Fermentation, including solid-state fermentation (SSF) and liquid-state fermentation (LSF), exerts distinct effects on polyphenolic compound accumulation in pulses. SSF predominates in most research about polyphenol enrichment through fermentation in pulses. Its low-moisture environment provides a relatively stable setting for microorganisms, not only favoring the maintenance of activity in polyphenol synthesis-related enzymes, but also promoting polyphenol accumulation. Molds, due to their ability to penetrate solid substrates with hyphae and form close contact with pulses, are commonly employed as SSF strains. This intimate contact facilitates thorough enzyme–substrate interactions, thereby enhancing the polyphenol yield.

However, the same solid-state fungal fermentation affects polyphenol content differently across pulses. Saharan et al. [13] utilized *Aspergillus awamori* to enrich polyphenols in diverse pulses, indicating that compared with the unfermented control, after 4 d of fermentation, the increase in TPC of black mung beans (2.5-fold) was significantly higher than that of green mung beans (1.7-fold). Similarly, Dhull et al. [14] demonstrated that fermentation enrichment of different lentil varieties using *Aspergillus awamori* increased the TPC by 78.8–122.8%. Furthermore, different fungal strains exhibited varying efficacy in enriching pulse polyphenols. It was found that *Aspergillus oryzae* ATCC 48012 significantly increased the quercetin (+62.3%) and kaempferol (+76.7%) content of broad beans, while ATCC 22959 specifically enriched the apigenin (2-fold increase) and *p*-coumaric acid (2.1-fold increase) [87]. In addition, in this study, ATCC 42222 exhibited the weakest enrichment capacity and demonstrated significant degradation of specific phenolic acids. The bacteria (*Bacillus* species, *Lactobacillales*) were also selected as strains for SSF enrichment of pulse polyphenols. Limón et al. [88] demonstrated that during SSF of common beans using *Bacillus* subtilis, soluble phenolic content (SPC) increased by 126% at 96 h. Similarly, Chen et al. [89] further elucidated strain specificity and synergistic effects. During the fermentation of lentil, *Lactobacillus paracasei* TK1501 demonstrated a superior TPC enrichment (8.3% increase) compared to *Lactobacillus plantarum* TK9 (3.4% increase), but co-fermentation of TK1501 with *Bacillus natto* achieved a 40.7% increase. Following the fermentation of mung beans using *Lactobacillus plantarum* CECT 748, TPC increased by 270% compared with the unfermented group [90]. Concurrently, several polyphenols (catechin and taxifolin) were produced.

For LSF, the high moisture content enhanced the mass transfer rates, facilitating efficient microbial metabolism and the activity of polyphenol-related enzymes. Chen et al. [89] employed LSF of lentils using *Lactobacillus paracasei* (TK1501), increasing the TPC from 3.02 mg/g (unfermented) to 3.89 mg/g (post-fermentation). Similarly, a study using HPLC quantification found that lactic acid *bacteria* fermentation led to a substantial increase (190%) in the TPC of peas, specifically raising the concentrations of caffeic acid, ellagic acid, and catechins, while also forming a new polyphenol (quercetin) [91]. However, most studies avoid LSF due to its susceptibility to pH reduction, as acidic conditions may compromise phenolic stability and glycosidase activity. Research has demonstrated that β-glucosidase produced by *Lactobacillus plantarum* inactivated at pH < 4, thereby inhibiting the release and conversion of polyphenol [92]. A study using *L. plantarum* LSF of common beans for 96 h revealed that pH decreased to 3.7–4.3, the SPC dropped from 20.7 to 17.8 mg/g, and hydroxybenzoic acids fell by more than 60% [88].

Significant progress has been made in research on the enrichment of polyphenols in pulses through fermentation, particularly in mung beans, lentils, and common beans. Studies have demonstrated the considerable potential of combining SSF and LSF with specific strains (*Aspergillus*, *Lactobacillus*, and *Bacillus*) to enhance polyphenol content and facilitate biotransformation [13,89,91]. However, systematic studies on azuki bean, chickpea, and lupins remain limited but can be conducted in future research.

**Table 4:** Effect of different enrichment techniques on the polyphenol content of pulses.

Enrichment Technologies	Pulses	Experimental Conditions	Increased TPC [93]	Key Findings	Reference
Fermentation	Mung bean	SSF, *Aspergillus awamori* MTCC 548, 6 d	+107.0%	↑ Gallic acid, ferulic acid	[13]
	Mung bean	LSF, *Lactobacillus plantarum* CECT 748, 48 h	+270.0%	↑ *p*-Hydroxybenzoic acid, *p*-hydroxyphenylacetic acid, *p*-coumaric acid (trans), apigenin-8-C-glucoside, apigenin-7-O-glucoside, eriodictyol-7-O-galactoside, catechin	[90]
	Azuki bean	LSF, *Lactobacillus paracasei* ASCC 279, 48 h	+71.4%	↑ Protocatechuic acid, quercetin, catechin	[94]
	Common bean	SSF, *Bacillus subtilis* CECT 39ᵀ ATCC 6051, 48 h	+95.6%	↑ *p*-Hydroxybenzoic acid, *p*-coumaric acid, ferulic acid, sinapic acid	[88]
	Common bean	LSF, *Lactobacillus paracasei* ASCC 279, 48 h	+66.7%	↑ Protocatechuic acid, ferulic acid, quercetin, catechin	[94]
	Pea	SSF, *Lactic Acid Bacteria*, 24 h	+190.9%	↑ Caffeic acid, rosmarinic acid, rutin, quercetin-3-glucoside, kaempferol-3-glucoside, quercetin, hesperetin, (–)-epigallocatechin, (+)-catechin	[91]
	Chickpea	SSF, *Cordyceps militaris* SN-18, 8 d	+80.7%	↑ Shikimic acid, chlorogenic acid, rutin, genistein, biochanin A	[95]
	Lentil Sapna	SSF, *Aspergillus awamori* MTCC 548, 6 d	+122.8%	↑ *p*-Coumaric acid, catechin	[14]
	Lentil	LSF, *Lactobacillus paracasei* TK1501	+28.8%	N.M.	
Germination	Mung bean	Dark, 5 d	+1697.0%	↑ Gallic acid, *p*-hydroxybenzoic acid, protocatechuic acid, vanillic acid, *p*-coumaric acid, ferulic acid, quercetin	[96]
	Azuki bean	Dark, 25 °C, 72 h	+38.6%	N.M.	[97]
	Common bean	Dark, 25 °C ± 2 °C, 6 d	+70.0%	↑ Gallic acid, syringic acid, 3-hydroxycinnamic acid (trans), ferulic acid, quercetin, catechin	[33]
	Pea	Dark, 25 °C ± 2 °C, 7 d	+55.9%	↑ Gallic acid, syringic acid, ferulic acid	
	Chickpea	Dark, 20 °C, 5 d	+240.0%	↑ Protocatechuic acid, (+)-catechin	[98]
	Lupin	Dark, 20 °C, 9 d	+184.7%	↑ *p*-Coumaric acid (trans), ferulic acid (trans), apigenin derivatives, luteolin derivatives, genistein derivatives	[99]
	Lentil	Dark, 25 °C, 5 d	+21.5%	N.M.	[100]
Germination + UAE	Mung bean	Dark, 37 ± 1 °C, 48 h; 300 W, 9 min; Ca^2+^	+143.3%	↑ Luteolin, quercetin-3-O-glucoside, astragalin, naringenin, epicatechin, taxifolin, malvidin, leucopelargonidin	[101]
	Common bean	Dark, 25 °C, 96 h; 360 W, 60 min, 40 kHz	+1065.0%	N.M.	[102]
Germination + UV	Mung bean	Dark, 20 °C, 60 h; UV-B, 20.5 μW/cm^2^, 2.5 h	+2.9%	N.M.	[103]
	Common bean	Light, 24.9 °C, 12 d; UV-C, 700 μW/cm^2^, 10 min	N.M.	↑ Gallic acid, syringic acid, vanillic acid, *p*-hydroxybenzoic acid, ferulic acid	[104]
	Lentil	Dark, 26 ± 2 °C; UV-A, 0.5 W/m^2^, 6 d	+8.1%	N.M.	[105]

Dark, all day; N.M., not mentioned; TPC, percentage increase compared to control (unfermented, ungerminated). ↑: Increased phenolic components compared to the control (unfermented, ungerminated).

### 5.2. Germination Enrichment

Germination enrichment activates key enzymes in the phenylpropanoid pathway (PAL, C4H, and 4CL) during seed sprouting, promoting the biosynthesis and release of polyphenols [106,107]. In a study by Guo et al. [108], mung beans exhibited a TPC of 966.4 ± 58.3 mg GA equiv/100 g DW at 9 d post-germination, representing a 4.5-fold increase over non-germinated mung beans. Similarly, after 120 h of germination, the TPC of the lentils increased by 21.5% compared with the untreated sample [100]. Furthermore, during the germination process, pulse seeds can activate secondary metabolic pathways and synthesize new polyphenols. In chickpeas, at 120 h post-germination, protocatechuic acid was detected for the first time, accompanied by a 240% increase in TPC [98].

During pulse germination, polyphenol content is influenced by factors such as germination duration, germination temperature, light exposure, and intensity. Huang et al. [109] observed that compared to ungerminated mung beans, germinated mung beans exhibited the TPC trend of initial increase followed by decline. Similarly, in the study by Tajoddin et al. [110], using the ALM-1 mung bean variety as an example, the TPC increased with germination duration, rising from 284 ± 0.02 mg GAE/100 g at 12 h to 354 ± 0.12 mg GAE/100 g at 48 h. This trend was also confirmed in peas, where the TPC increase was higher at 48 h (77.8%) than at 24 h (22.2%) [111]. Germination temperature was positively correlated with TPC. The TPC of chickpeas germinated at 30 °C (1.53 mg GE/g) was higher than that at 20 °C (1.06 mg GE/g), possibly due to the increased enzyme activity promoting the release of bound polyphenols [112]. However, a higher fermentation temperature is not necessarily better, it must be maintained within an optimal range. The TPC of mung beans at 35 °C (2.4 mg/g) was lower than that at 30 °C (2.7 mg/g), possibly due to the decreased thermal stability of polyphenols at higher temperatures [106]. Research indicated that mung beans germinating in darkness exhibited TPC concentrations 1.46-to-1.78-fold higher than those under light exposure [16,96]. The light-induced reduction in TPC is mainly due to the activation of polyphenol oxidases, accelerating polyphenol degradation, coupled with increased ROS production that may contribute to their oxidative damage. Furthermore, the choice of extraction solvent was crucial for the thorough extraction of polyphenols following germination. When polyphenols were extracted from germinated chickpeas, the TPC obtained with acetone (193.7 mg/kg) was significantly higher than that obtained with methanol (75.6 mg/kg) or hexane (66.9 mg/kg) [113].

Studies showed that, in addition to increasing polyphenol content, germination could effectively reduce the concentrations of antinutrients such as phytic acid in various pulses. Compared with heat treatment, germination was more effective in reducing the phytic acid content in pulses [111]. For example, germination for 48 h reduced the phytic acid content in lentils by up to 75.7% [111]. Similarly, the phytic acid content in mung beans decreased by 38.9% following germination, which was significantly superior to heat treatment (roasting, 22.2%; autoclaving, 31.3%) [114]. This reduction was significant because antinutrients interfered with the bioavailability of polyphenols, thereby affecting their interaction with the intestinal environment. Consequently, germination was not only a strategy for increasing polyphenol content but also enhanced the gut health benefits of pulse polyphenols.

Based on the above analysis, the TPC of mung beans, azuki beans, common beans, peas, chickpeas, and lentils increases significantly after germination. However, systematic studies on pulses such as broad beans and lupins remain limited, representing an area worthy of further investigation.

### 5.3. Other Physical Auxiliary Enrichment Techniques

Ultrasound, as an emerging non-thermal technology, has been extensively demonstrated to enhance the polyphenol content in pulses when applied during germination synergistically. One study indicated that at 48 h post-germination in mung beans, the traditional germination group exhibited a TPC of 6.1 mg/g [101]. In contrast, the ultrasonic pretreated germination group reached 7.63 mg/g. Ampofo et al. [102] reported that ultrasonic treatment (360 W, 60 min) followed by 96 h of germination increased the total phenolic acids, flavonoids, and anthocyanins in common bean sprouts by 6.6- to 11.7-fold compared to the control. Furthermore, treatment with an appropriate ultrasonic frequency can effectively increase polyphenol concentrations after germination. When azuki beans were treated with ultrasound at 28, 40, and 80 kHz prior to germination, the TPC in the 40 kHz group (8.44 mg GAE/g FW) was higher than that in the 28 kHz (6.62 mg GAE/g FW) and 80 kHz groups (5.89 mg GAE/g FW) [115]. Surprisingly, combining ultrasonication with other exogenous ions or reagents significantly enhanced polyphenol content in pulses. Yu et al. [101] reported that combined ultrasonic–Ca^2+^ treatment increased the TPC of germinating mung beans at 48 h to 11.58 ± 0.39 mg/g, versus 7.63 ± 0.10 mg/g with ultrasound alone, due to Ca^2+^ activation of polyphenol synthesis enzymes via calcium signaling. Combined ultrasonication and γ-aminobutyric acid (GABA) treatment of mung beans for 48 h increased free flavonoids and free polyphenols by 11.45% and 3.13% over ultrasonication alone [116].

Ultraviolet stress primarily exerts its effects through the regulation of polyphenol synthesis genes [117]. Exposure of mung bean sprouts to UV-B for 2.5 h led to a peak TPC, 2.94% higher than that of the untreated group [103]. Park et al. [105] demonstrated that UV-A-treated green lentils exhibited a significantly higher TPC at 6 d post-germination (3.91 mg GAE/g DW) compared to the dark-treated control group (3.32 mg GAE/g DW). UV wavelength and exposure time are crucial for the accumulation of polyphenols in germinated pulses. UV-C irradiation of common bean seeds for 10–15 min significantly enhanced the accumulation of specific phenolic acids in seedlings, with distinct profiles in roots and foliage [104]. For instance, gallic acid reached a peak of 6.65 µg/mL at 10 min, while chlorogenic acid peaked at 23.74 µg/mL after 15 min. Similarly, in lentils, continuous UV-B irradiation caused ferulic acid in the FG cultivar to peak at 37.73 μg/mL on day 6. In contrast, under identical intensities, UV-A treatment resulted in a higher TPC (3.59 mg GAE/g DW) in the LG variety than UV-B (3.30 mg GAE/g DW) [105].

## 6. Conclusions and Further Perspectives

Pulse polyphenols primarily consist of phenolic acids, flavonoids, and tannins, predominantly located in the seed coat, with significantly higher concentrations in dark-colored varieties compared to light-colored ones. Their gut health benefits depend on maintaining homeostasis by regulating the balance of the gut microbiota, improving intestinal barrier function, and exerting antioxidant and anti-inflammatory effects. Technologically, SSF outperforms LSF in enriching polyphenols due to greater polyphenol stability, while combining germination with physical stresses such as ultrasound or UV radiation significantly boosts polyphenol content. However, research on pulse polyphenols and their applications remains limited. Regarding their effects on gut health, most existing studies rely on in vitro or animal models, necessitating further clinical validation and research into their mechanisms of action. Furthermore, future research should focus on optimizing technical parameters and integrating multiple techniques to assess the impact of processing on the structural activity of pulse polyphenols, as well as any potential risks.

Nevertheless, based on current evidence, this review mainly focused on the in vitro or animal studies, with a lack of clinical trials in humans. The doses used in animal models may not be directly translatable to human intake. Furthermore, systematic research on certain pulses, such as broad beans and lupins, remained limited. Future research should prioritize clinical validation, elucidation of mechanisms, and the integration and optimization of enrichment technologies to fully explore the potential of pulse polyphenols for gut health.

## Data Availability

The original contributions presented in this study are included in the article. Further inquiries can be directed to the corresponding authors.
